# Computed tomography–based radiomic analysis for prediction of treatment response to salvage chemoradiotherapy for locoregional lymph node recurrence after curative esophagectomy

**DOI:** 10.1002/acm2.13434

**Published:** 2021-10-06

**Authors:** Liang Gu, Yangchen Liu, Xinwei Guo, Ye Tian, Hongxun Ye, Shaobin Zhou, Fei Gao

**Affiliations:** ^1^ Department of Radiation Oncology Taixing People's Hospital Tai Xing Jiangsu Province China; ^2^ Department of Radiation Oncology The Second Affiliated Hospital of Soochow University Su Zhou Jiangsu Province China

**Keywords:** chemoradiotherapy, computed tomography, esophageal squamous cell carcinoma, predictor, radiomics analysis

## Abstract

**Objective:**

To investigate the capability of computed tomography (CT) radiomic features to predict the therapeutic response and local control of the locoregional recurrence lymph node (LN) after curative esophagectomy by chemoradiotherapy.

**Methods:**

This retrospective study included 129 LN from 77 patients (training cohort: 102 LN from 59 patients; validation cohort: 27 LN from 18 patients) with postoperative esophageal squamous cell carcinoma (ESCC). The region of the tumor was contoured in pretreatment contrast‐enhanced CT images. The least absolute shrinkage and selection operator with logistic regression was used to identify radiomic predictors in the training cohort. Model performance was evaluated using the area under the receiver operating characteristic curves (AUC). The Kaplan–Meier method was used to determine the local recurrence time of cancer.

**Results:**

The radiomic model suggested seven features that could be used to predict treatment response. The AUCs in training and validated cohorts were 0.777 (95% CI: 0.667–0.878) and 0.765 (95% CI: 0.556–0.975), respectively. A significant difference in the radiomic scores (Rad‐scores) between response and nonresponse was observed in the two cohorts (*p* < 0.001, 0.034, respectively). Two features were identified for classifying whether there will be relapse in 2 years. AUC was 0.857 (95% CI: 0.780–0.935) in the training cohort. The local control time of the high Rad‐score group was higher than the low group in both cohorts (*p* < 0.001 and 0.025, respectively). As inferred from the Cox regression analysis, the low Rad‐score was a high‐risk factor for local recurrence within 2 years.

**Conclusions:**

The radiomic approach can be used as a potential imaging biomarker to predict treatment response and local control of recurrence LN in ESCC patients.

## 1 INTRODUCTION

Esophageal cancer ranks seventh in terms of incidence and sixth in overall mortality worldwide.^1^ It ranks fifth in China and is the fourth leading cause of cancer‐related deaths in the country, and 90% of esophageal carcinoma patients are diagnosed with esophageal squamous cell carcinoma (ESCC).^2^ Surgical resection is the primary treatment for potentially curable esophageal cancer. However, lymph node (LN) recurrence after surgery is one of the main reasons of treatment failure.^3^ The prophylactic therapy is found effective in the T3–4 or N1–3 postresection pathological stage.^4^, ^5^, ^6^, ^7^ However, some patients who failed to receive adjuvant treatment due to developing postoperative complications or refusal to take treatment reported LN recurrence soon after surgery. T1–2 N0 M0 cancer patients were regularly followed up after surgery; some of them developed locoregional LN recurrence within 2 years because of tumor heterogeneity and underestimated staging.^8^ The salvage chemoradiotherapy (CRT) remarkably improved the survival rate of these patients, and the patients who were sensitive to CRT had a longer survival time than the patients insensitive to CRT.^9^, ^10^, ^11^ Prediction of the efficacy of CRT on recurrent LNs (RLNs) remains a challenge.

Radiomics refers to the high‐throughput extraction of quantitative data from medical images and then mining of correlations between different features for the diagnosis/prognosis of the disease. Since it was first proposed in 2012,^12^, ^13^ radiomics has attracted widespread attention of researchers worldwide owing to a noninvasive, quantitative, and low‐cost approach. It is promising in diagnosing different tissue characteristics, tumor staging, and treatment response.^14^, ^15^ It can serve as a digital and modeling method to predict the effect of radiotherapy (RT) or chemotherapy on a tumor. It has been investigated in lung cancer, prostate cancer, breast cancer, and other tumors.^16^, ^17^, ^18^, ^19^, ^20^ Several studies have focused on predicting preoperative staging of esophageal cancer and evaluating the response to CRT,^21^, ^22^, ^23^ though fewer studies have used radiomics to predict the response in locoregional RLN after esophagectomy treatment by salvage radiochemotherapy.

In this study, we aimed to build a radiomic signature to assess therapeutic response and local control of salvage RT or CRT for LN recurrence after curative esophagectomy.

## 2 MATERIALS AND METHODS

### 2.1 Patients

A total of 129 metastatic LNs from 77 patients with postoperative ESCC were investigated in the current retrospective study. The study was approved by the ethics committee. Prior written informed consent was obtained from each of the study participants. The salvage CRT or RT was administered to the ESCC patients with LN recurrence postsurgery from January 2015 through December 2016. We followed the following inclusion criteria for the selection of participants:

1. Patients received curative esophagectomy and pathologically confirmed SCC, had not received chest RT treatment;
2. The LN recurrences were located within the bilateral supraclavicular region and mediastinum; the diagnostic approaches for lymph metastasis included physical examinations, B‐mode ultrasound, computed tomography (CT) of the supraclavicular and thoracic region, positron emission tomography (PET)‐CT, and histological confirmation through biopsy;
3. No clear contraindications to RT and chemotherapy, and no distant metastases such as heart, brain, lung, and bone; and
4. Detailed follow‐up information of patients within 3 years available.

A total of 85 patients were recruited. The exclusion criteria were as follows:

1. The information on follow up was insufficient (*n* = 5);
2. The RT treatment was administered after esophagectomy (*n* = 2); and
3. The pathology was not SCC (*n* = 1).

Therefore, according to the 7th edition of American Joint Committee on Cancer (AJCC) staging, 129 LNs of 77 patients were included in the study, of which 47 patients had a single LN metastasis, and 30 patients reported two or more LN metastases. The patients were randomly assigned to the training cohort and validation cohort in a ratio of about 10:3. In the training cohort, models were built and then were validated in the validation cohort. The clinic characteristics of patients in both cohorts are presented in Table 1.

**TABLE 1:** Characteristics of patients in training and validation cohorts

Characteristics	Training cohort	Validation cohort	*p*	Summary
Number of patients	59	18		77
Number of LN	102	27		129
Gender				
Female	9	5	0.295	14
Male	50	13		63
Age, median (range)	64 (46–79)	64 (50–74)	0.433	64 (46–79)
Number of LN per patient				
Single	36	11	0.778	47
≥2	23	7		30
T stage				
T1+2	26	11	0.159	37
T3+4	33	7		40
N involved				
N+	29	8	0.792	37
N−	30	10		40
Median LN recurrence time (month (range))	11 (1–72)	18 (5–60)	0.138*	11 (1–72)
POCT				
Yes	24	7	0.559	31
No	35	11		46
CRT for LN				
Yes	40	9	0.262	49
No	19	9		28
Median radiation dose, Gy (range)	60 (50–64)	60 (50–64)	0.194	60 (50–64)
Treatment response				
Response (CR + PR)	75	18	0.148	93
Nonresponse (SD + PD)	27	9		36
Median control time of LN, month (95% CI)	13.0 (11.5–14.4)	12.0 (9.3–14.7)	0.109*	13.0 (11.9–14.0)

*Note*: χ^2^ test and Fisher's exact test for categorized variables, two‐sample *t*‐test for continues variables.

Abbreviations: CR, complete response; CRT, concurrent chemoradiotherapy; LN, lymph node; PD, progressive disease; POCT, postoperative adjuvant chemotherapy; PR, partial response; SD, stable disease; −, metastasis negative; +, metastasis positive.

* Log‐rank test.

### 2.2 Chemoradiotherapy

All patients received three‐dimensional conformal radiation therapy. The gross tumor volume (GTV) was defined as RLNs identified by CT scans or PET/CT. The clinical target volume (CTV) was defined as GTV with a 0.5–1.5 cm expansion range. The planning tumor volume was ascertained by adding 0.5 cm radially to the CTV. A total dose of 50–64 (median, 60) Gy was delivered as 2 Gy per fraction 5 days a week. Also, 28 patients received radiation therapy alone and 49 patients received CRT of TP (paclitaxel, 135 mg/m^2^ on day 1 and cisplatin, 25 mg/m^2^ on days 1–3, 28 days per cycle). According to toxicity levels, the dose adjustment was implemented in the second chemotherapy cycle in 10 patients.

### 2.3 Treatment evaluation and follow up

One month after the completion of the treatment, the therapeutic response was assessed using CT image with contrast, according to the Response Evaluation Criteria in Solid Tumors 1.1.^24^ Patients with complete response (CR, the disappearance of all target lesions confirmed at ≥4 weeks) or partial response (PR, ≥30% decrease from the short diameter of the LN in the CT images) were considered responders, while those with progressive disease (PD, ≥20% increase in the short diameter of the LN) or stable disease (SD, neither PR nor PD criteria met) were classified as nonresponders. After the completion of therapy, a physical examination at every 1‐ or 3‐month interval was carried out using ultrasonography or CT. Local‐regional failure time was calculated from the end of the first CRT or RT to the time of the second recurrence.

### 2.4 Image acquisitions and tumor segmentation

All patients underwent contrast‐enhanced CT of the chest performed using a 64‐channel multidetector CT scanner (LightSpeed VCT, GE Medical Systems, Milwaukee, WI, USA) in our hospital between January 2015 and December 2016. The acquisition parameters were as follows: 120 kV; 160 mAs; 0.4 s or 0.5 s rotation time; detector collimation, 64 × 0.625 mm or 64 × 1.25 mm; the field of view, 350 mm × 350 mm; and matrix, 512 × 512. The contrast‐enhanced CT was performed after a 25 s delay following intravenous administration of 85 ml of iodinated contrast material (Iohexol injection, Yangtze River Pharmaceutical Group, Jiangsu, China) at a rate of 3.0 ml/s with a pump injector. All the CT images were reconstructed with a standard kernel. These CT images were retrieved from the picture archiving and communication system.

Regarding the RT plan in the treatment planning system and AJCC 7th edition staging, two radiation oncologists with >5 years of experience in interpreting esophageal carcinoma radiology outlined the metastatic LNs in the clavicle, the mediastinal lymphatic drainage area, and performed manual segmentation of the metastatic LNs on each patient's CT images. An open‐source and free software platform for biomedical research called 3D‐slicer (version 4.8.1, https://www.slicer.org/)^25^ was employed for this task. These regions of interest (ROI) were used in subsequent feature extraction for further analysis.

### 2.5 Feature extraction in radiomics

Pyradiomics, an extension in 3D‐slicer, is an open‐source Python package for the extraction of radiomic features from CT imaging.^26^ There were 106 features: 13 shape‐based, 18 first‐order, 24 gray‐level co‐occurrence matrix (GLCM), 16 gray‐level size zone matrix (GLSZM), 5 neighboring gray‐tone difference matrix, 14 gray‐level dependence matrix, and 16 gray‐level run length matrix (GLRLM) features (Table S1). The ROI were manually delineated slice by slice by two expert radiologists (Readers 1 and 2, with clinical experience of 10 and 8 years, respectively, in esophageal cancer RT). Reader 1 delineated the ROI again a month later. Reader 2 manually sketched the ROI only once. The interclass correlation coefficient (ICC) was used to determine the agreement in feature values between the observers. In our study, radiomic features with ICC greater than 0.75 were extracted, and reader 1 delineated the ROI for the first time for further study.

### 2.6 Statistical analysis

All statistical analyses were performed on R software (version 3.5.3, http://www.r‐project.org/). The difference in the categorical variables between the two groups was assessed with the two‐sample *t*‐test, chi‐square test, or Fisher's exact test, as appropriate. The Kaplan–Meier method and log‐rank test were used to estimate disease‐free survival (DFS). Multivariate analyses were performed using the Cox proportional‐hazards model. The least absolute shrinkage and selection operator (LASSO) with logistic regression was applied to identify optimal predictors in the training cohort by the “glmnet” package. The “ggplot2” and “pROC” packages were employed to draw receiver operating characteristic curves and evaluate the model performance by the area under the receiver operating characteristic curves (AUC). The “survival” and “survminer” packages were used for survival analysis and to draw survival cures. A two‐sided *p*‐value of <0.05 was considered statistically significant.

## 3 RESULTS

### 3.1 The radiomic model for therapeutic response

**FIGURE 1:**
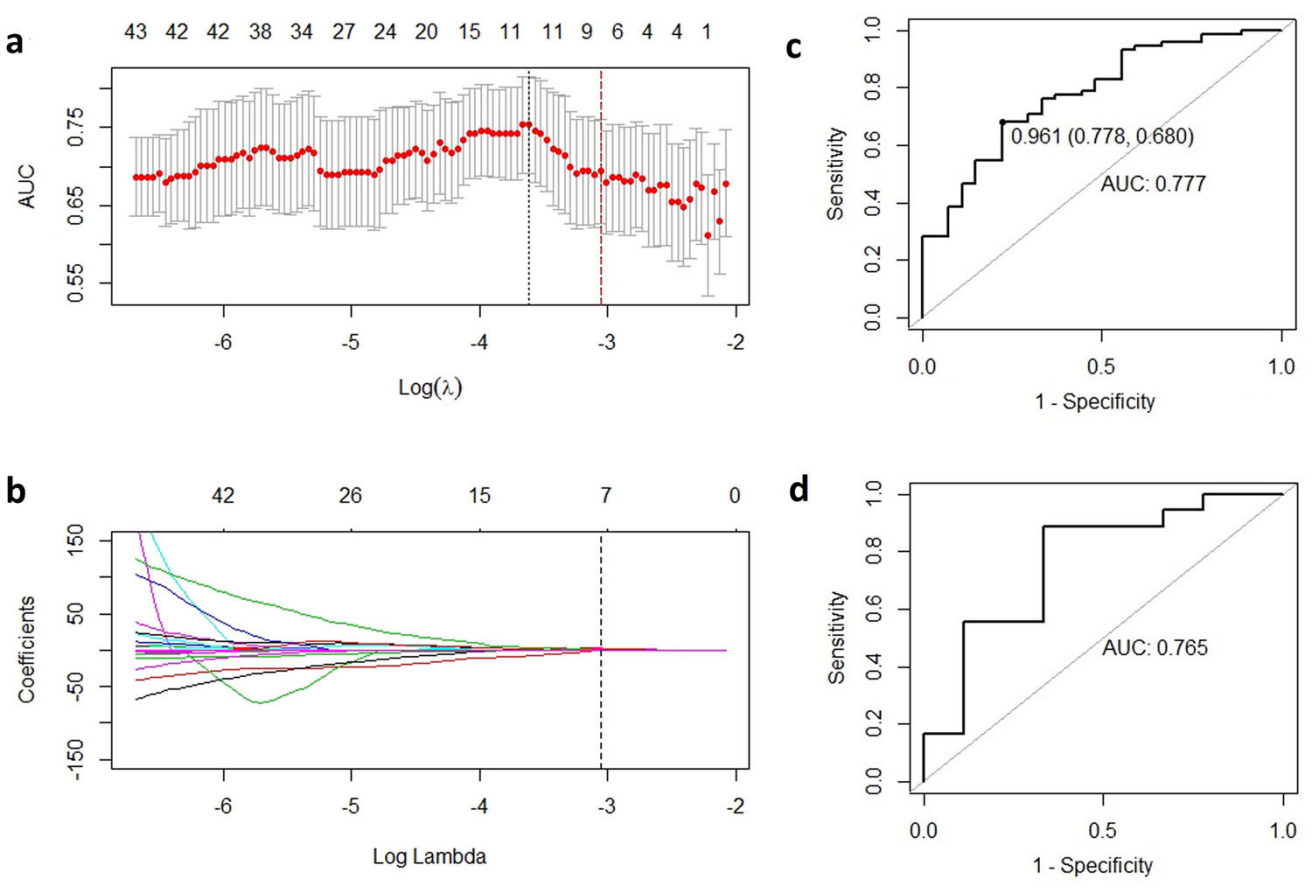
Radiomics feature selection using the least absolute shrinkage and selection operator logistic regression model; (a) the cross‐validation curve; the area under the receiver operating characteristic curve was plotted versus log (Lambda). Red dotted vertical lines were drawn at the optimal value by using 10‐fold cross‐validation and the 1 standard error of the minimum criteria (the 1‐SE criteria). (b) Coefficient profiles of 106 radiomics features; the vertical dotted line was drawn at the value selected in (a). The Lambda value of 0.0471, with log (Lambda) = −3.056 was chosen, and seven nonzero coefficients were selected. (c, d) Receiver operating characteristic curves for Rad‐score in training and validated cohorts; when the cutoff value was 0.961, the specificity was 0.778 and the sensitivity was 0.680

The response rate of the metastatic LNs in this study was 73.5% (75/102) in the training cohort and 66.7% (18/27) in the validation cohort (*p* = 0.148). No clinical differences were found between the training cohort and the validation cohort (Table 1). The LASSO (54) with logistic regression was used to select the most significant radiomic features for therapeutic response in the training cohort (Figure 1), and ultimately seven features were identified from 106 features (the ICCs of all features were greater than 0.75). The radiomic score (Rad‐score) was calculated by these radiomic features (Table 2). The Rad‐scores in the training cohort were 1.243 ± 0.433 and 0.698 ± 0.433 for response and nonresponse, respectively (*p* < 0.001). A statistically significant difference in Rad‐score in the validated cohort was also observed (1.303 ± 0.566 vs. 0.816 ± 0.451, *p* = 0.034). The AUCs in the training cohort and validated cohort were 0.777 (95% CI: 0.667–0.878) and 0.765 (95% CI: 0.556–0.975), respectively (Figure 1).

**TABLE 2:** Coefficients and features of seven radiomic signatures for response

Coefficients	Features
−3.215941 (β_0_)	Constant
−0.4714669 (β_1_)	Shape sphericity (χ_1_)
1.627270 (β_2_)	GLCM—inverse variance (χ_2_)
0.03268378 (β_3_)	First order—interquartile range (χ_3_)
0.01575666 (β_4_)	First order—90 percentile (χ_4_)
0.003081561 (β_5_)	First order—kurtosis (χ_5_)
2.5310139 (β_6_)	GLRLM—short run emphasis (χ_6_)
2.987626^10^–9^ (β_7_)	GLSZM—large area high gray‐level emphasis (χ_7_)

Abbreviation: GLSZM, gray‐level size zone matrix.

### 3.2 The radiomic model for the 2‐year local control rate

**FIGURE 2:**
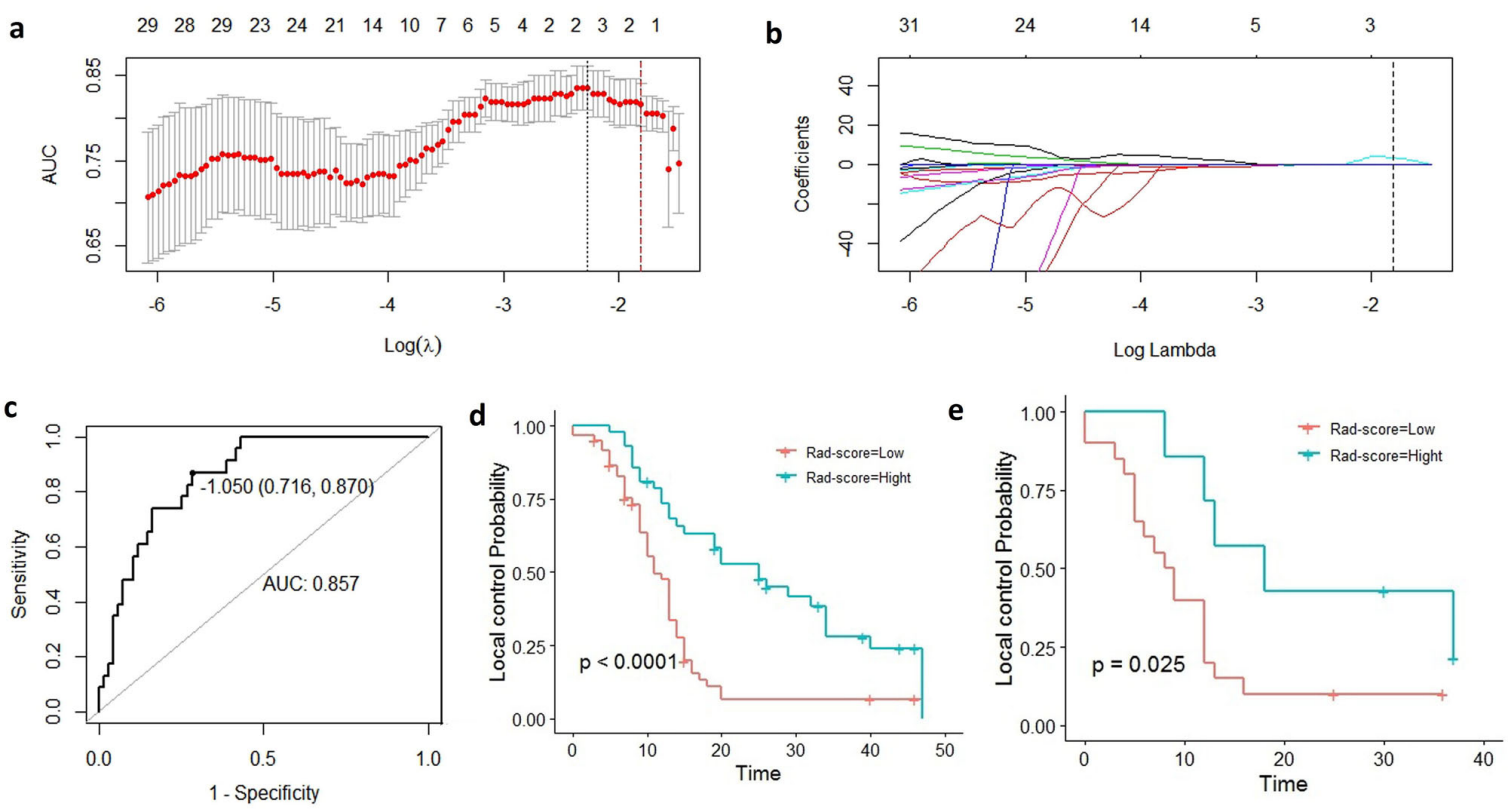
Radiomics feature selection using the least absolute shrinkage and selection operator logistic regression model; (a) the area under the receiver operating characteristic cross‐validation curve was plotted versus log (Lambda). Red dotted vertical lines were drawn at the optimal value by using fivefold cross‐validation and the 1‐SE criteria. (b) Coefficient profiles of 106 radiomics features; the vertical dotted line was drawn at the value selected in (a). The Lambda value of 0.1638, with log (Lambda) = −1.809 was chosen, and two nonzero coefficients were selected. (c) Receiver operating characteristic curves for Rad‐score‐2‐year in the test set composed by ninety lymph; (d, e) lymph node local control curve with Rad‐score‐2‐year (Kaplan–Meier); *p*‐values were 0.001 and 0.025 in the training and the validation groups, respectively (long‐rank)

In the training cohort, the 12 metastatic LNs taken from seven patients who died within 2 years were excluded because no local recurrence was observed. Finally, 90 LNs were used to build a radiomic model to predict 2 years of local recurrence. The 2‐year control rate was 25.5% (23/90). First, we considered whether recurrence occurred within 2 years as a binary variable; then we used LASSO (9) to select the most significant radiomic features for classifying whether there would be relapse. At last, two features were identified (Figure 2), and the Rad‐score of the 2‐year local control rate (Rad‐score‐2‐year) was determined using the following equation:

(1) $$\begin{array}{ccc} & & \mathrm{Rad}-\mathrm{score}-2-\mathrm{year}=-3.281461437\\ & & +0.002394247*(\mathrm{Firstorder}-\mathrm{Interquartile}\mathrm{Range})\\ & & +3.434397106*(\mathrm{Glrlm}-\mathrm{Run}\mathrm{Percentage}) \end{array}$$

The AUC of the Rad‐score‐2‐year in the training cohort composed of 90 LNs was 0.857 (95% CI: 0.780–0.935). When the cutoff value was −1.015, the specificity was 0.716 and the sensitivity was 0.870. Rad‐score‐2‐year greater than the cutoff value was presumed to be a high Rad‐score; otherwise, it was a low Rad‐score. Considering Rad‐score high and low as a layering factor, the local control rate of the high Rad‐score group was higher than that of the low Rad‐score group both in the training and the validation cohorts, with *p*‐values <0.001 and 0.025, respectively (Figure 2).

In order to further determine the relationship between the Rad‐score and the local control of LNs for 2 years, 129 LNs (including those in the training cohort and verification cohort) were further divided into a 2‐year local control group and a 2‐year local uncontrolled group according to the local control situation within 2 years. On comparison of clinical characteristics of the two groups, we observed that the T staging, radiation dose, treatment response, and Rad‐score‐2‐year were statistically different (*p* = 0.005, 0.01, <0.001, <0.001, respectively) (Table S2), and concurrent CRT was given at a critical state (*p* = 0.071). After Cox regression analysis of the above five factors, we found that only the treatment response and Rad‐score‐2‐year were statistically different (<0.001 and <0.001, respectively, Table 3). A LN that had a low Rad‐score‐2‐year and was nonresponsive to treatment was more likely to experience recurrence within 2 years.

**TABLE 3:** Cox regression analysis for 2‐year local control

Factor	HR	95% CI	*p*‐value
Treatment response			
CR + PR	1		
SD + PD	6.01	3.48–10.4	<0.001
Rad‐score‐2‐year			
High	1		
Low	2.21	1.40–3.50	<0.001

Abbreviation: SD, stable disease.

## 4 DISCUSSION

Some studies have predicted the therapeutic response using radiomics in esophageal cancer.^23^, ^27^, ^28^, ^29^, ^30^ Hou et al.^30^ noticed that five features of contrast‐enhanced CT images discriminated nonresponders from responders (AUCs from 0.686 to 0.727) in esophageal carcinoma treated by CRT. Yang et al.^23^ postulated three predictive models using radiomic parameters for pathologic CR (pCR) after neoadjuvant chemotherapy and radiation therapy (nCRT) in ESCC (AUC = 0.84–0.86 in training cohorts and 0.71–0.79 in testing cohorts). Beukinga et al.^31^ constructed a prediction model by combining ^18^F‐FDG PET radiomics and clinical T stage in predicting pCR to nCRT, where AUC was 0.81. Many studies have highlighted the primary lesions of the esophagus, but this study focused on the recurrence of LNs postsurgery in esophageal cancer. We used a CT radiomic model to predict its response to RT or CRT. Seven radiomic features were selected by LASSO with 10‐fold cross‐validation, including one shape‐based feature, one GLCM, one GLRLM, one GLSZM, and three first‐order features. The AUCs were 0.777 and 0.765 in the training and validated cohorts, respectively, similar to the previous findings, although the microenvironment and heterogeneity of tumor metastasis are different from those of the primary lesion and the response to treatment is also different.^32^ This study establishes that CT‐based radiomics can still predict its therapeutic response. Local recurrence within 2 years is still one of the principal reasons for progression in these patients.^10^, ^33^ Hence, we used the radiomic model to predict the 2‐year local control rate. At last, two features were chosen by LASSO with fivefold cross‐validation, and AUC was 0.857 in the training cohort. Due to the low 2‐year local control rate (25.93%) in this study and the small number of cases (*n* = 27) in the verification cohort, only five LNs could be controlled over 2 years. The AUC method was not a good way to calculate the predictive ability of the model in the validated cohort. Therefore, we used the level of Rad‐score‐2‐year as a risk factor to create a local recurrence time curve, and the *p*‐values were less than 0.05 in the training cohort and the validated cohort. Cox regression analysis revealed that low Rad‐score‐2‐year was a high‐risk factor for local recurrence in 2 years in the current study. We used different methods to show that the Rad‐score generated by the radiomic model could predict the treatment response and local recurrence of mediastinal metastatic LNs after RT or CRT. For patients with poor results, other treatment options such as immunotherapy^34^ were considered.

Several studies have revealed contradictory results on whether tumor size or volume could be considered as a risk predictor for treatment response.^10^, ^23^, ^35^, ^36^ Yang et al.^23^ observed that tumor volume could not predict pCR in any of the patients in their cohort, but they documented that surface volume ratio (SVR, surface area to volume ratio; a lower SVR indicates a more compact shape) might provide more information about pCR than tumor volume did. Our study also noticed that shape sphericity (a measure of the roundness of the shape of the tumor region relative to a sphere; Table 2) was related to the response of metastatic LNs to RT or CRT. Tumor heterogeneity was correlated with tumor proliferation, necrosis, and hypoxia, and may be correlated to poor response and worse prognosis.^37^ The texture‐based features exhibited the spatial arrangement of voxels and disclosed the change of local intensity in the tumor region.^38^ Emerging evidence has suggested that image‐based quantification of tumor heterogeneity may provide important information for predicting response to treatment and prognosis in esophageal cancer patients.^31^, ^39^, ^40^ The GLCM entropy was the most often reported radiomic feature, reflecting the local randomness (irregularity) within the image, and where low GLCM entropy represented a more homogeneous texture.^40^ The model of therapeutic response in the current study contained GLCM‐inverse variance, another measure of the local homogeneity of an image. Yip et al.^41^ noticed the pretreatment and posttreatment standard deviation of a histogram showed a borderline association with pathological tumor response. A proportional change in skewness < 0.39 was associated with improved survival. Hou et al.^30^ highlighted that histogram skewness, histogram kurtosis, and GLSZM long‐zone emphasis discriminated nonresponders from responders using an artificial neural network–derived prediction model in 49 patients. In our study, the prediction model for 2‐year local control rate contained two texture‐based features, first‐order‐interquartile range (the range of gray values) and GLRLM‐run percentage (the coarseness of the texture by taking the ratio of several runs and number of voxels), both indicating the heterogeneity of the tumor. We constructed the multiparameter prediction model to improve the predictive value of the multiple feature combination; however, nine features were found related to tumor heterogeneity, with only one feature reflecting the shape of the tumor. It suggested that the heterogeneity of the tumor was related to treatment effect and survival. This was similar to many other CT‐based radiomic models.^23^, ^28^

This study had certain limitations. First, we applied only basic radiomic features, overlooking the Laplacian of Gaussian filter approach, which could have been able to reduce image noise and highlight different anatomical spatial scales within the tumor.^29^ Second, this was a retrospective study with a small sample size, and the data were derived from a single institution. Our results need to be validated in multiple centers with a larger and prospective patient cohort in the future.

## 5 CONCLUSIONS

Overall, in this study, we constructed a CT‐based radiomic model to predict the efficacy of salvage RT or CRT for LN recurrence after curative esophagectomy. The proposed model demonstrated a strong prognostic value. Therefore, as a noninvasive and quantitative method, the radiomic approach can be employed as the potential imaging biomarker for clinical practice in the prediction of treatment response and local control to salvage RT or CRT for LN recurrence in patients with ESCC. It is suggested to further study and fine tune the radiomic model by this approach to improve predictive performance through larger, wider multicentric metastudies.

## AUTHOR CONTRIBUTIONS

Liang Gu, Hongxun Ye, and Ye Tian designed the study. Liang Gu and Hongxun Ye prepared figures and wrote the manuscript. Xinwei Guo, Shaobin Zhou, Yangchen Liu, and Fei Gao collected the follow‐up data. Liang Gu and Xinwei Guo performed the statistical analysis. All authors reviewed and approved the final manuscript.

## ETHICS APPROVAL

The study was approved by the ethics committee of Taixing People's Hospital (LS2021011) and was performed in accordance with the standards of the Declaration of Helsinki. Written informed consent was obtained from all participants in the study. The manuscript has not been published elsewhere.

## CONFLICT OF INTEREST

The authors have no conflict of interest to declare.

## Supporting information



Supporting information

## Data Availability

The data that support the findings of this study are available from the corresponding author upon reasonable request.
