# Supplementary material for: Computed tomography–based radiomic analysis for prediction of treatment response to salvage chemoradiotherapy for locoregional lymph node recurrence after curative esophagectomy
Source: J Appl Clin Med Phys. 2021 Oct 6;22(11):71–9. doi: 10.1002/acm2.13434 (PMC8598151; doi:10.1002/acm2.13434)
Supplement: Supplementary file 1 — Supporting information [file ACM2-22-71-s001.pdf]

Supplementary Materials

Table S1：The radiomics features of 3D-slicer(version 4.8.1)

shape	Maximum3DDiameter	gldm	GrayLevelVariance
	Maximum2DDiameterSlice		HighGrayLevelEmphasis
	Sphericity		DependenceEntropy
	MinorAxis		DependenceNonUniformity
	Elongation		GrayLevelNonUniformity
	SurfaceVolumeRatio		SmallDependenceEmphasis
	Volume		SmallDependenceHighGrayLevelEmphasis
	MajorAxis		DependenceNonUniformityNormalized
	SurfaceArea		LargeDependenceEmphasis
	Flatness		LargeDependenceLowGrayLevelEmphasis
	LeastAxis		DependenceVariance
	Maximum2DDiameterColumn		LargeDependenceHighGrayLevelEmphasis
	Maximum2DDiameterRow		SmallDependenceLowGrayLevelEmphasis
Glcm			LowGrayLevelEmphasis
	JointAverage	First-order	InterquartileRange
	SumAverage		Skewness
	JointEntropy		Uniformity
	ClusterShade		Median
	MaximumProbability		Energy

Glcm	Idmn	First-order	RobustMeanAbsoluteDeviation
	JointEnergy		MeanAbsoluteDeviation
	Contrast		TotalEnergy
	DifferenceEntropy		Maximum
	InverseVariance		RootMeanSquared
	DifferenceVariance		90Percentile
	Idn		Minimum
	Idm		Entropy
	Correlation		Range
	Autocorrelation		Variance
	SumEntropy		10Percentile
	MCC		Kurtosis
	SumSquares		Mean
	ClusterProminence	glszm	GrayLevelVariance
	Imc2		ZoneVariance
	Imc1		GrayLevelNonUniformityNormalized
	DifferenceAverage		SizeZoneNonUniformityNormalized
	Id		SizeZoneNonUniformity
	ClusterTendency		GrayLevelNonUniformity
glrlm	ShortRunLowGrayLevelEmphasis	glszm	LargeAreaEmphasis
	GrayLevelVariance		SmallAreaHighGrayLevelEmphasis

	LowGrayLevelRunEmphasis		ZonePercentage
	GrayLevelNonUniformityNormalized		LargeAreaLowGrayLevelEmphasis
	RunVariance		LargeAreaHighGrayLevelEmphasis
	GrayLevelNonUniformity		HighGrayLevelZoneEmphasis
	LongRunEmphasis	glszm	SmallAreaEmphasis
glrlm	ShortRunHighGrayLevelEmphasis		LowGrayLevelZoneEmphasis
	RunLengthNonUniformity		ZoneEntropy
	ShortRunEmphasis		SmallAreaLowGrayLevelEmphasis
	LongRunHighGrayLevelEmphasis		Coarseness
	RunPercentage		Complexity
	LongRunLowGrayLevelEmphasis	ngtdm	Strength
	RunEntropy		Contrast
	HighGrayLevelRunEmphasis		Busyness
	RunLengthNonUniformityNormalized		

Table S2 :Characteristics of patients in 2-year local control and uncontrol cohorts

Characteristics	control	uncontrol	*P*
Number of LN	28	101	
Gender			
Female	6	21	1.000
Male	22	80	
Age(Median (range)）	62（53-74）	65（46-79）	0.211
T stage			
T1+2	20	40	0.005
T3+4	8	61	
N involved			
N＋	10	53	0.138
N－	18	48	
Median LN recurrence time (month (range)）	12（1－48）	11（2－72）	0.793*
POCT			
YES	10	42	0.666
NO	18	59	
CRT for LN			
YES	23	64	0.071

NO	5	37	
Median radiation dose(Gy(range)）	60(54-64)	60（50-64）	0.039
Treatment response			
response （CR+PR)	28	65	＜0.001
nonresponse （SD+PD)	0	36	
Rad-score			
Hight	23	26	＜0.001
Low	5	75	

Note: The 12 metastatic lymph nodes taken from seven patients in uncontrol cohort were not observed the recurrence time because of the patients died within 2 years.

χ^2 test and Fisher’ s exact test for categorized variables; two-sample t-test or wilcoxon test for continues variables

* log-rank test

Abbreviations: LN, lymph node; +, metastasis positive; - metastasis negative，

CRT:Concurrent chemoradiotherapy

POCT: Postoperative adjuvant chemotherapy
